# An Electrochemical DNA Microbiosensor Based on Succinimide-Modified Acrylic Microspheres

**DOI:** 10.3390/s120505445

**Published:** 2012-04-27

**Authors:** Alizar Ulianas, Lee Yook Heng, Sharina Abu Hanifah, Tan Ling Ling

**Affiliations:** 1 School of Chemical Sciences and Food Technology, Faculty of Science and Technology, Universiti Kebangsaan Malaysia, 43600 Bangi, Selangor, Malaysia; E-Mails: alizar_chem@yahoo.co.id (A.U.); sharina@ukm.my (S.A.H.); 2 Industrial Chemistry Programme, Faculty of Industrial Sciences & Technology, Universiti Malaysia Pahang, 26300 Gambang, Kuantan, Pahang, Malaysia; E-Mail: babybabeoo@gmail.com

**Keywords:** DNA microbiosensor, acrylic microspheres, hybridization, photopolymerization, succinimide

## Abstract

An electrochemical microbiosensor for DNA has been fabricated based on new acrylic microspheres modified with reactive *N*-acryloxysuccinimide (NAS) functional groups. Hydrophobic poly(*n*-butylacrylate-*N*-acryloxysuccinimide) microspheres were synthesized in an emulsion form with a simple one-step photopolymerization technique. Aminated DNA probe was attached to the succinimde functional group of the acrylic microspheres via covalent bonding. The hybridization of the immobilized DNA probe with the complementary DNA was studied by differential pulse voltametry using anthraquninone-2-sulfonic acid monohydrate sodium salt (AQMS) as the electroactive hybridization label. The influences of many factors such as duration of DNA probe immobilization and hybridization, pH, type of ions, buffer concentrations, ionic strength, operational temperature and non-complementary DNA on the biosensor performance were evaluated. Under optimized conditions, the DNA microbiosensor demonstrated a linear response range to target DNA over a wide concentration range of 1.0 × 10^−16^ and 1.0 × 10^−8^ M with a lower limit of detection (LOD) of 9.46 × 10^−17^ M (R^2^ = 0.97). This DNA microbiosensor showed good reproducibility with 2.84% RSD (relative standard deviation) (n = 3). Application of the NAS-modified acrylic microspheres in the construction of DNA microbiosensor had improved the overall analytical performance of the resultant DNA microbiosensor when compared with other reported DNA biosensors using other nano-materials for membranes and microspheres as DNA immobilization matrices.

## 1. Introduction

The immobilization method and the matrix used for DNA probe immobilization are important in designing a DNA biosensor especially for achieving high sensitivity, selectivity and stablility [1–4]. Immobilizing of DNA probes onto an immobilization matrix or electrode requires strong binding between the probe and the immobilization matrix without affecting the chemical properties of the DNA probe [5]. Various methods for immobilization of DNA probes have been reported such as physical and electrochemical adsorptions [6–8], electrochemical entrapment [9] and covalent binding [10–12]. Sorption and entrapment methods are simple immobilization methods; however they produce weak molecular bonds between the biological molecules and the immobilization matrix. This results in the immobilized molecules leaching out easily and reduces the shelf life and stability of the biosensor. In general, covalent bonding and the biotin-avidin methods are found to be more suitable for DNA probe immobilization where one end of the DNA probe is attached, leaving the other end free for hybridization with target DNA to form a double-stranded DNA (dsDNA) [13]. In addition, the covalent bonding formed is stronger and will not interfere with the chemical behaviour of the DNA probe and thus it is an efficient method to yield high performance DNA biosensors in terms of sensitivity, selectivity and stability [14,15]. DNA probe immobilization via covalent bonding methods often involves the use of linker functional groups for DNA probe immobilization. Some examples are the functional groups of succinimide [16–19], aldehyde [20], and maleimido-based reactive groups [21,22]. Thus, using an immobilization matrix modified with such functional groups would create a useful immobilization surface for covalent immobilization of DNA probes.

The type of matrix for DNA probe immobilization also plays an important role affecting the performance of an electrochemical DNA biosensor. Nanoparticles used for immobilization matrices, e.g., nanoparticles and microspheres, can contribute to better performance of the resulting biosensor when compared with membrane matrices. This is due to the larger surface area of the three-dimensional structure of nanoparticles [23,24] when compared with the two-dimensional structure of a membrane. With larger surface area, DNA probe binding capacity can be improved and this further improves the DNA biosensor performance. Several polymer membranes [3,4,25–29] and microspheres, such as metal based gold and Fe_2_O_3_ [30–34] microspheres have been employed for DNA biosensor construction, but microsphere-based DNA biosensors exhibited better performance compared with polymer membrane matrices.

**Figure 1.**
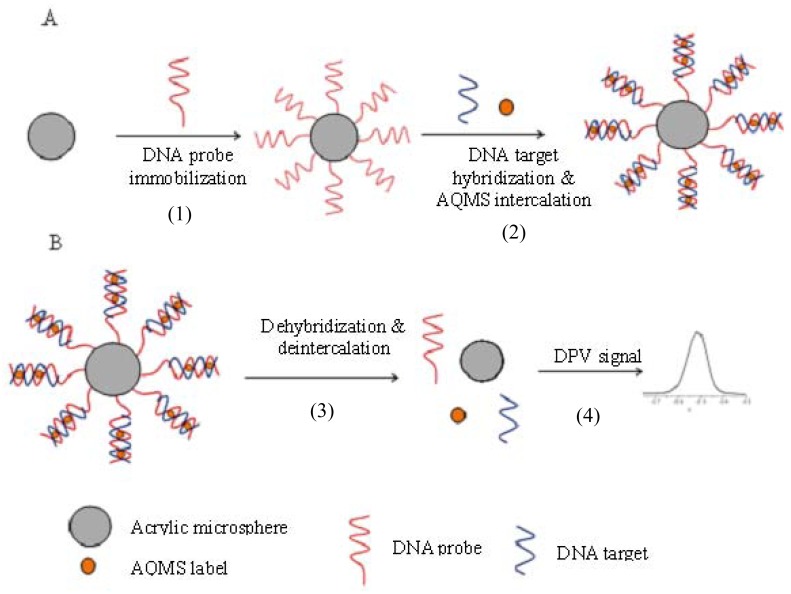
Scheme for the immobilization and labeling of hybridization of DNA using poly(nBA-NAS) microspheres (**A**) and the releasing of AQMS label for DPV detection (**B**).

In this research, acrylic polymer microspheres modified with succinimide functional groups via N-acryloxysuccinimide (NAS) moieties was used as the matrix for DNA probe immobilization. As previously reported [35,36], the succinimide functional group can react with amine functional groups to form a covalent bond. The incorporation of a NAS functionality into acrylic microspheres for DNA microbiosensor application is a new idea that provides advantages of a simple preparation method where the spheres can be synthesized and functionalised via a one-step procedure using photopolymerisation in a short duration (several minutes). In addition, the microspheres have the advantage of small size and provide a large surface area for DNA probe immobilization, thus reducing the barrier to diffusion for reactants and products. This enables the improvement in the biosensor performance in terms of shorter response times and wider linear response range, which will be demonstrated in the work reported here. The mechanism of construction of DNA microbiosensing system using NAS functionalized acrylic microspheres is depicted in Figure 1.

## 2. Experimental Section

### 2.1. Instrumentation

All electrochemical measurements were performed with DPV (AutoLab potentiostat) in a measurement cell containing 4.5 mL of 0.05 M K-phosphate buffer at pH 7.0. The electrochemical system consists of a gold electrode (GE), a carbon pencil counter electrode and Ag/AgCl reference electrode. A Scanning Electron Microscope (SEM, LEO 1450VP) was used to determine the size and distribution of the acrylic microspheres.

### 2.2. Chemicals

The reagents 2-2-dimethoxy-2-phenylacetophenone (DMPP), 1,6-hexanadiol diacrylate (HDDA) and sodium dodecyl sulphate (SDS) were supplied by Fluka, Aldrich and Systerm, respectively. N-acryloxysuccinimide (NAS) and anthraqinone-2-sulfonic acid monohydrate sodium salt (AQMS) were obtained from Acros. The 20-base pair single stranded DNA was purchased from Sigma-Aldrich. The DNA sequences for this work were similar to those used in the previous study by Wong *et al.* [37]. These oligonucleotide DNA sequences are as follows:

Probe DNA: 5′ GGGGCAGAGCCTCACAACCT (AmC_3_)
Target DNA: 5′ AGGTTGTGAGGCTCTGCCCC
Non-complementary DNA: 5′ GGATGGACGAAGCGCTCAGG

Oligonucleotide stock solution (100 μM) was prepared in TE buffer solution containing 10 mM Tris-HCl and 1 mM ethylenediaminetetra-acetic acid (EDTA) at pH 7.7 and stored under −20 °C when not in use. Dissolution of oligonucleotide stock solution was carried out using 0.05 M K-phosphate buffer pH 7.0. Stock solution of 1 mM AQMS was prepared in 0.05 M K-phosphate buffer (pH 7.0).

### 2.3. Synthesis of NAS-Modified Acrylic Microspheres

A mixture of nBA monomers (7 mL), SDS (0.01 g), HDDA (450 μL), DMPP (0.1 g), NAS (6 mg) and H_2_O (15 mL) was sonicated for 10 min. The resulting emulsion solution was then photocured for 600 s with ultraviolet radiation of a wavelength of approximately 250–350 nm under a continuous nitrogen gas flow. Poly(nBA-NAS) microspheres formed were then collected by centrifugation at 4,000 rpm for 30 min and later washed three times in 0.05 M K-phosphate buffer (pH 7.0), followed by air drying.

### 2.4. Fabrication of DNA Biosensor by Immobilization of DNA Probe on NAS-Modified Acrylic Microspheres

About 200.0 mg of poly(nBA-NAS) microspheres were added to 5.0 μM DNA probe solution and kept at 4 °C for 24 h in order to immobilize the probes onto the microspheres. Subsequently, the microspheres were washed with 0.05 M K-phosphate buffer (pH 7.0) to remove the unbound DNA probes from the microspheres. To test the success of the DNA probe immobilization, hybridization studies were carried out by using a complementary DNA target. The acrylic microspheres immobilized with DNA probes [DNA-poly(nBA-NAS)] were first immersed in a solution containing 5.0 μM complementary DNA target and 1.0 mM AQMS. The mixture was then incubated at 4 °C for 24 h. After that, the microspheres were collected and washed with 0.05 M K-phosphate buffer at pH 7.0 to remove the free DNA target and unbound AQMS. The resultant microspheres attached with the hybridized DNA and intercalated AQMS were sonicated in 4.5 mL of 0.05 M K-phosphate buffer (pH 7.0) to dislodge the hybridized DNA and released the AQMS label from the hybridized DNA. The DPV of the AQMS release from sonication was scanned at the potential range of −0.75 V to −0.25 V using a gold electrode as working electrode, a carbon pencil counter electrode and Ag/AgCl reference electrode.

### 2.5. Effects of Reaction Medium on DNA Microbiosensor Response

The response of DNA microbiosensor was examined based on the effect of various parameters on the hybridization of the immobilised DNA probe. This was performed in the present of different cations (Na^+^, K^+^, Mg^2+^, Ca^2+^, Al^3+^), varying pH from pH 6.0–8.5 and in different Na-phosphate buffer concentrations between 0.002 M to 1.0 M. Ionic strength effect on hybridization of DNA probe and biosensor response was also examined by varying the NaCl concentration over the range of 0.02–2.0 M at pH 7.5.

### 2.6. Effect of Duration of Probe Immobilization and Hybridization Temperature on DNA Microbiosensor Response

The durations of probe immobilisation and temperature of hybridization with target DNA could influence the microbiosensor response. For these studies, immobilization of DNA probe was performed in 0.05 M K-phosphate buffer pH 7.0, whilst for hybridization of DNA target, it was carried out in 0.25 M Na-phosphate buffer (pH 7.5) in the presence of 0.5 M Na^+^ ion at 4 °C. Both the duration of probe immobilisation and hybridization were performed over a time period of 1–24 h. For temperature effect on DNA hybridization, the temperature was varied from 4–45 °C over a period of 20–160 min. In these studies, the effect on the linear response range and lower detection limit of the biosensor under each condition was examined.

## 3. Results and Discussion

### 3.1. Confirmation of DNA Probe Immobilization on NAS-Modified Acrylic Microspheres

**Figure 2.**
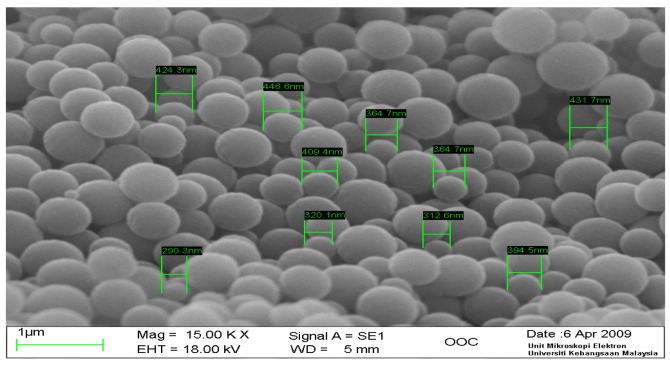
SEM image of acrylic polymer microspheres.

A typical scanning electron micrograph image of the as prepared acrylic microspheres (Figure 2) demonstrated that the size of the microspheres was of a diameter approximately in the μm range, with a rather homogenous size distribution. These microspheres that have been modified with NAS functional group were used for DNA probe immobilization via the aminated end of the DNA probe.

**Figure 3.**
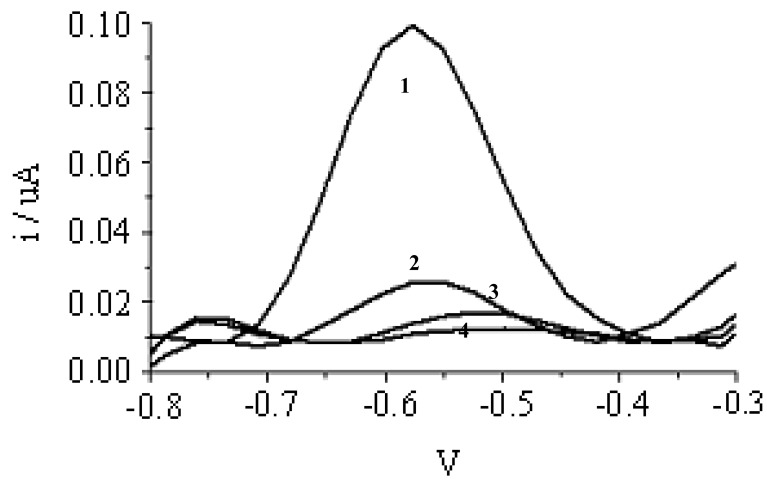
Differential pulse voltamogram for AQMS after the DNA-modified acrylic microspheres were exposed to complementary DNA (1), non-complementary DNA (2), no DNA (3) and blank acrylic microsphere (no DNA immobilised) (4) (Working electrode used was a gold electrode, the counter electrode was a carbon pencil and Ag/AgCl as a reference electrode).

Figure 3 shows the DPV peak at −0.5 V for AQMS that has been intercalated into DNA probe immobilized on poly(nBA-NAS) microspheres after hybridization with complementary DNA. However, the current at peak −0.5V was small and non-observable for events that did not involved hybridization such as in the presence of non-complementary DNA, in the absence of DNA and blank microspheres (no immobilised DNA probes; Experiments 2–4). The higher current response observed for Experiment 1 compared with Experiment 2, *i.e.*, when non-complementary DNA was introduced, indicated that the immobilized DNA probe was selective towards complementary DNA and it was indicated by AQMS intercalation into dsDNA formed as has been previously reported [3,38–40]. Control Experiments 3 and 4 were also performed to determine whether any non-specific adsorption of AQMS on ds-DNA probe or blank acrylic microsphere. The relatively small currents observed in Experiments 3 and 4 compared with Experiment 1 implied that the non-specific adsorption of AQMS was negligible. The slight adsorption of AQMS onto DNA probe (ssDNA) was consistent with observation previously reported [41]. The highest current observed for the complementary DNA (Experiment 1) thus indicated hybrization where intercalation of AQMS had occurred in dsDNA formed on the microsphere surface.

### 3.2. Effect of DNA Probe Loading on DNA Microbiosensor Response

**Figure 4.**
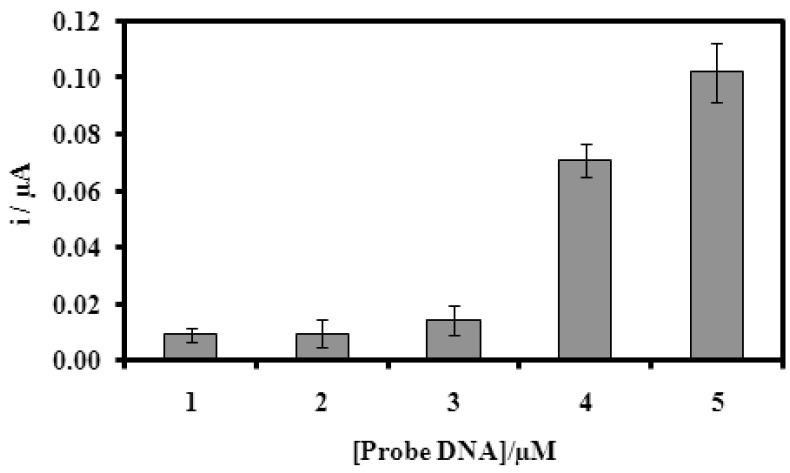
The effect of DNA probe loading on the hybridization current indicated by AQMS. Hybridization was performed with 5.0 μM complementary DNA in 0.05 M phosphate buffer (pH 7.0) containing 1.0 mM AQMS. DPV measurements were performed with gold electrode as a working electrode, carbon pencil as the counter electrode and Ag/AgCl as a reference electrode.

Figure 4 represents the DNA microbiosensor response with various concentrations of DNA probe immobilized onto the acrylic microspheres after hybridization with complementary DNA and followed by intercalation of AQMS. DNA microbiosensor response increased proportionally with increasing concentration of DNA probe immobilised. This suggests that the capacity of immobilized DNA probe to hybridize with complementary DNA has increased with an increase in DNA probe attached onto the acrylic microspheres. The dependence of DNA hybridization on DNA probe concentration has also been reported previously [15,42,43].

### 3.3. Dependence of Biosensor Response on pH and Ionic Strength

**Figure 5.**
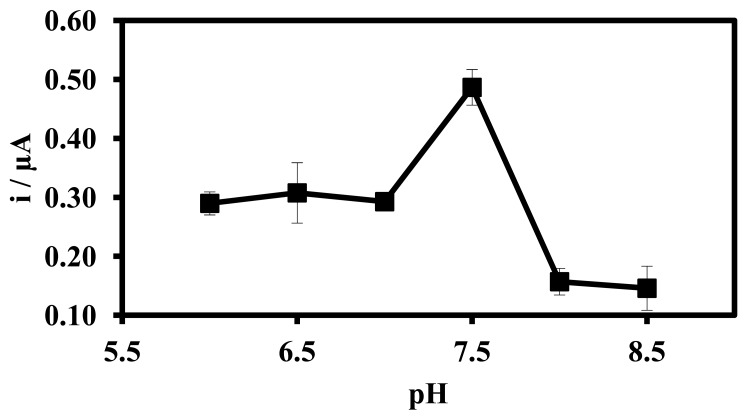
The pH effect on the microbiosensor response as indicated by the hybridization current with complementary DNA in Na-phospahte buffer solution. The concentration of DNA probe used for immobilisation and complementary DNA was 5.0 μM. (DPV measurements were performed with gold electrode as a working electrode, carbon pencil as the counter electrode and Ag/AgCl as a reference electrode).

Figure 5 illustrates the pH effect on DNA hybridization response of the biosensor. The DPV current increased abruptly at pH 7.5, after which a sharp decline in current response was observed under more alkaline conditions. The increased response of the DNA microbiosensor at pH 7.5 indicates that more DNA probes were hybridized with complementary DNA at this pH. Previous studies [44] have reported that the rate of DNA hybridization reaction can be influenced by the pH of the solution. At a more acidic environment, the protonation reaction of the phosphodieter of the DNA can reduce the solubility of the DNA molecule, which eventually decreases the DNA hybridization [3]. Under a more basic medium, DNA hybridization also decreased and hence the response of DNA biosensor was also lower [45]. Therefore, the optimize pH of DNA hybridization was selected at pH 7.5 using Na-phosphate buffer solution for subsequent biosensor studies.

**Figure 6.**
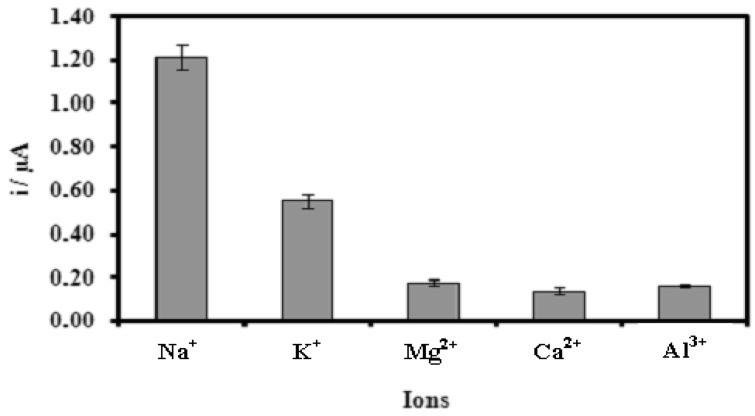
The effect of various cations on the DNA hybridization current of the microbiosensor. The concentration of cation used was 1.0 M. DNA hybridization was performed with 5.0 μM complementary DNA in 0.25 M Na-phosphate buffer (pH 7.5) containing 1.0 mM AQMS. (DPV measurements were performed with gold electrode as a working electrode, carbon pencil as the counter electrode and Ag/AgCl as a reference electrode).

Positively charged ions such as Li^+^, Na^+^, K^+^, and Mg^2+^ ions can interact with the negatively charge phosphodiester chain of the DNA. This ionic reaction will neutralize the charge of the DNA molecule and thus decreases the electrostatic repulsions between DNA molecules. The absent of electrostatic repulsion eases the DNA hybridization reaction [46–48]. Figure 6 depicts the effect of some cations on the DNA hybridization reaction of the biosensor. The DNA hybridization reaction rate increased in the present of positively charged ion in the order of Na^+^ > K^+^ > Al^3+^≈Ca^2+^≈Mg^2+^. The presence of Ca^2+^ and Al^3+^ ions had reduced the DNA hybridization compared with that of Na^+^ and K^+^ ions because the ionic interactions of Ca^2+^ and Al^3+^ ions with phosphate ions from the buffer lead to formation of insoluble phosphate compounds. Thus, this reduces the ionic content of the medium and increases the electrostatic repulsion between DNA molecules. Under such conditions, hybridization is more difficult to achieve and the biosensor response declined. The higher DNA hybridization current as indicated by the biosensor response in the presence of Na^+^ ion was due to the smaller size and stronger affinity of Na^+^ ion towards DNA phosphodieter chain to reduce the electrostatic repulsion between DNA molecules as compared with K^+^ and Mg^2+^ ions. As the use of Na^+^ ion demonstrated better biosensor signal than K^+^, Mg^2+^, Ca^2+^ and Al^3+^ ions, therefore Na^+^ ion was used in further DNA biosensor studies based on modified acrylic microspheres.

**Figure 7.**
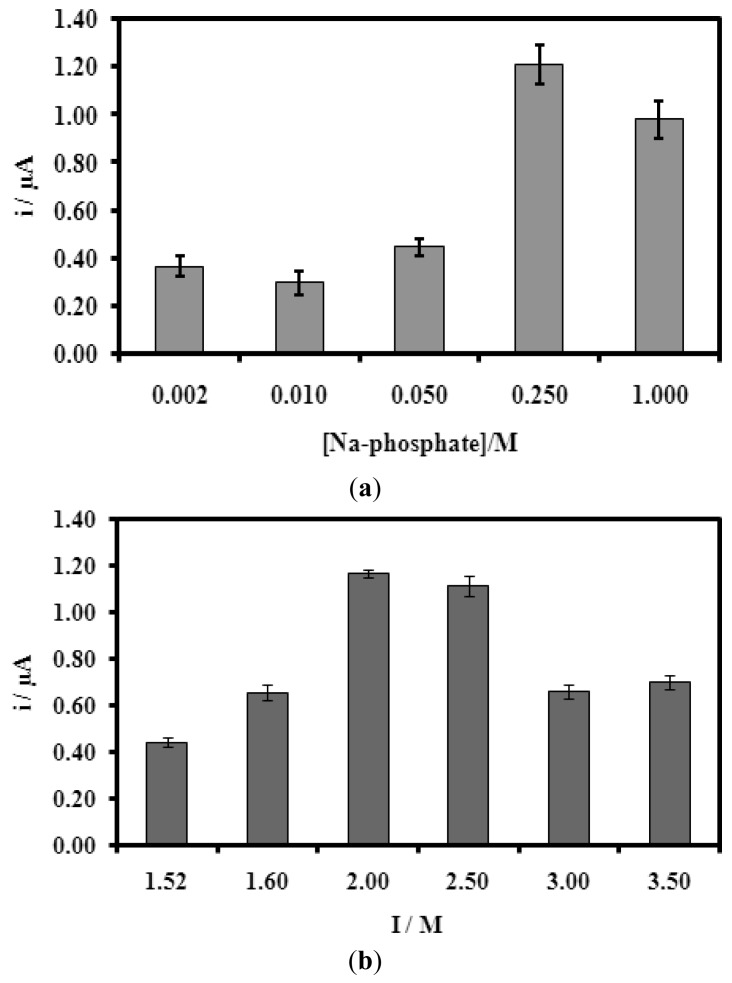
The effect of (**a**) varying of Na-phosphate buffer concentration (pH 7.5) and (**b**) different ionic strength (I) on the biosensor response. 5.0 μM of complementary DNA was used for the hybridization process.

In Figure 7(a,b), both Na-phosphate buffer (pH fixed at 7.5) concentration and ionic strength have effect on the biosensor response. In both cases, there is an optimum value where the biosensor gave the highest current response or the highest degree of hybridization. Thus, 0.25 M Na-phosphate buffer (pH 7.5) and 2.0 M ionic strength (from NaCl) were found to be optimum for the biosensor response. This may be explained by at certain amount of ionic content of the solution, the electrostatic repulsion between DNA molecules decreases and thus improving the DNA hybridization reaction. In the presence of too low or too high ionic content, the presence of electrostatic repulsion becomes dominant and the hybridization of DNA molecules become difficult [46].

### 3.4. Influence of the Duration of DNA Immobilization on Microbiosensor Response

**Figure 8.**
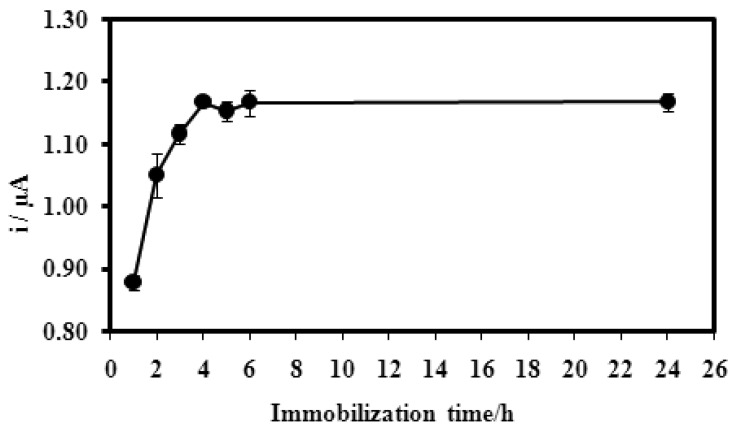
Effect of duration of DNA probe immobilisation on biosensor response. The duration of immobilization of DNA probes onto acrylic microspheres in 0.05 M phosphate buffer (pH 7.0). The hybridization with complimentary DNA did in 0.25 M Na-phosphate buffer (pH 7.5) and 2.0 M ionic strength. The concentrations of DNA probe and DNA target were 5.0 μM.

For the duration of immobilisation, the microbiosensor response showed a current increase from 1.0–4.0 h of immobilisation time, after which there was no obvious change in the current measured (Figure 8). Longer immobilization times resulted in the higher amount of DNA probes immobilized onto the microspheres. After 4.0 h of exposure to the DNA probes, the active sites of microspheres are presumably fully attached with DNA probes.

### 3.5. Hybridization Duration and Temperature Effects on Microbiosensor Response

**Figure 9.**
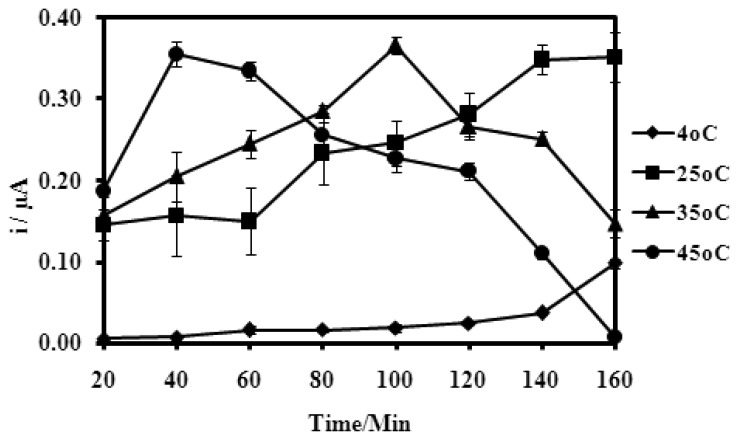
The microbiosensor current response towards different temperatures over a time duration of 160 min (Hybridization in 2.0 μM complementary DNA, 0.25 M Na-phosphate buffer at pH 7.5 and 2.0 M ionic strength).

Temperatures appear to affect the time taken for the maximum DPV current response of the microbiosensor. When temperatures were raised from 4 °C and 45 °C, the time taken for maximum DNA hybridization reduces to just about 40 min at 45 °C (Figure 9).

However at 4 °C, there was no obvious increase in the current response, even up to 140 min hybridization time. The increase in DNA hybridization rate at higher temperatures was attributed to a greater mass transfer rate and rate of reaction of DNA molecules, as well as an increase in the solubility of the DNA molecules under higher temperature conditions [46]. The microbiosensor response also dependent on the duration allowed for hybridization to occur. The increase in DPV current with hybridization time indicated that more DNA hybridization reactions were occurring [32]. The optimum microbiosensor response (Figure 9) is dependent on the temperature and reached at a shorter time span for higher hybridization temperatures. The length of DNA hybridization time obtained herein is similar to that reported elsewhere [41]. Even at room temperature of 25 °C, the current response of the microbiosensor is large enough for further biosensor studies.

### 3.6. Performance of the DNA Microbiosensor Based on NAS-Modified Acrylic Microspheres

**Figure 10.**
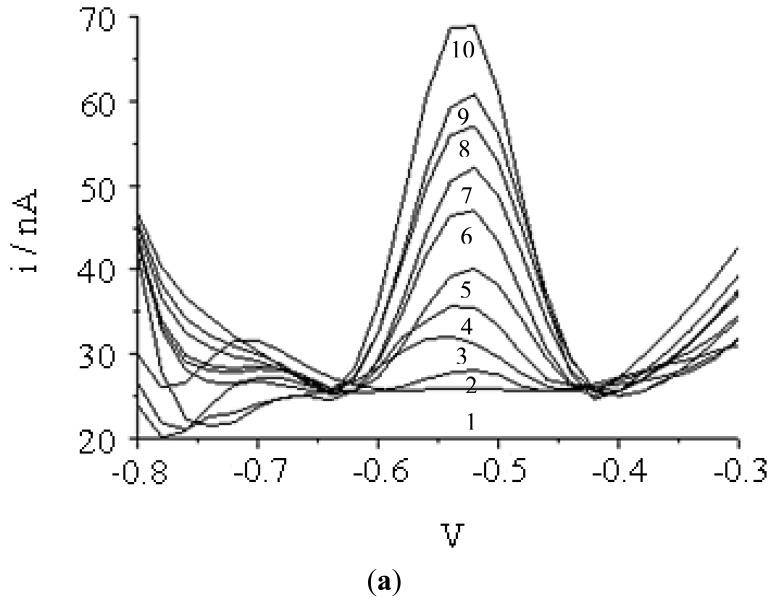

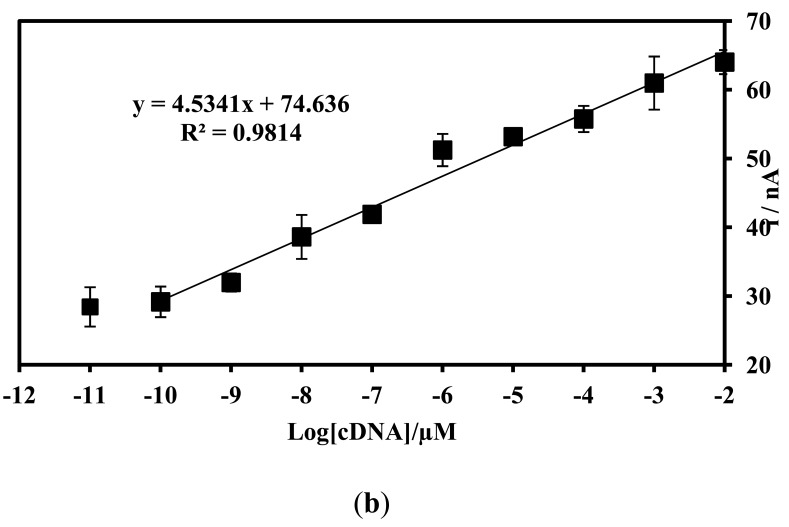
(**a**) Differential pulse voltammograms of AQMS intercalated onto dsDNA after hybridization with different concentrations of complementary DNA: (1) 1.0 × 10^−17^, (2) 1.0 × 10^−16^, (3) 1.0 × 10^−15^, (4) 1.0 × 10^−14^, (5) 1.0 × 10^−13^, (6) 1.0 × 10^−12^, (7) 1.0 × 10^−11^, (8) 1.0 × 10^−10^, (9) 1.0 × 10^−9^ and (10) 1.0 × 10^−8^ M; (**b**) The linear response range of the DNA microbiosensor response. DNA Hybridization was performed at optimum condition of 0.25 M Na-phosphate buffer (pH 7.5) and 2.0 M ionic strength and at 25 °C with 30 min of hybridization time.

Figure 10 demonstrates the effect of different complementary DNA concentrations on the biosensor response. The current response increases with increasing DNA concentrations indicates more DNA hybridization. This current response was linear towards the DNA concentration from 1.0 × 10^−16^ and 1.0 × 10^−8^ M with a LOD of 9.46 × 10^−17^ M. Using two DNA concentrations of 1.0 × 10^−13^ M and 1.0 × 10^−10^ M, the DNA microbiosensor yielded a good reproducibility with the RSD value of 2.86% (n = 3). The performance of the DNA microbiosensor based on NAS-modified acrylic microspheres yielded a wider linear response range and lower detection limit when compared with a DNA microbiosensor employing the same sequence of DNA probes self-assembled on a mercaptol modified gold electrode, which yielded a linear response range from 5.0 × 10^−10^ to 1.0 × 10^−6^ M with detection limit at 10^−12^ M level [37].

### 3.7. Comparison with Other Reported DNA Microbiosensors

A comparison of the DNA microbiosensor based on NAS-modified acrylic microspheres with other electrochemical DNA biosensors reported in the literature was carried out (Table 1). For most DNA biosensors based on electrochemical transduction, membranes doped with nanomaterials were often used. For example, the use of chitosan films doped with carbon nanotubes or CeO_2_ [3,28].

In the literature, DNA microbiosensors based on microspheres normally require the microspheres to be immobilized in some form of matrices, e.g., polyaniline nanofibers doped with Fe_2_O_3_ microspheres or Au microspheres supported by polyaniline nanofiber. This clearly demonstrates the advantage of the acrylic microspheres where the acrylic polymeric microspheres can function as a DNA microbiosensor alone after attachment of DNA probe. Overall, the DNA microbiosensor from this work using acrylic microspheres has shown large improvement in terms of linear response range and detection limit where the detection limit is at least 1,000 times lower that those DNA biosensors designed from nanomaterials or other microsphere materials.

## 4. Conclusions

Acrylic microspheres for use as a DNA immobilization matrix has been synthesized using the methods of emulsion and photopolymerization simultaneously. Covalent immobilization of DNA probes onto the microspheres was possible via succinimide functional groups incorporated during the synthesis step. The designed DNA microbiosensor based on acrylic microspheres showed good performance with a wider linear response range and the capability of detecting DNA targets at fM concentration. In addition, there is no specific adsorption of AQMS on the acrylic microspheres. The performance of the new DNA biosensor using succinimide functional group-modified acrylic microspheres was better compared with the other electrochemical transduction-based DNA biosensors.

## Figures and Tables

**Table 1.** A comparison of the performance of electrochemical DNA biosensors employing various materials for DNA immobilisation.

**Immobilization matrix**	**Transduction method**	**Dynamic range (M)**	**Detection limit (M)**	**Reproducibility (RSD%)**	**Ref.**
Acrylic microspheres	Amperometry	1.0 × 10^−16^ to 1.0 × 10^−8^	9.46 × 10^−17^	2.86	This work
CeO_2_/chitosan composite film	Amperometry	1.59 × 10^−11^ to 1.16 × 10^−7^	1.0 × 10^−11^	4.04	[3]
Poly-l-lysine films	Electrochemical impedance	1.0 × 10^−12^ to 1.0 × 10^−7^	3.1 × 10^−13^	3.16	[25]
Chitosan/nano-V_2_O_5_/MWCNTs	Amperometry	1.0 × 10^−11^ to 1.0 × 10^−6^	1.76 × 10^−12^	3.0	[28]
Poly-2,6-pyridine-dicarboxylic acid film	Amperometry	1.0 × 10^−10^ to 1.0 × 10^−5^	12.4 × 10^−11^	-	[29]
Fe_2_O_3_ microspheres/polyaniline nanofibers	Electrochemical impedance	1.0 × 10^−13^ to 1.0 × 10^−7^	2.1 × 10^−14^	3.58	[32]
Polyaniline nanofiber/ Au microspheres	Amperometry	1.0 × 10^−13^ to 1.0 × 10^−6^	1.9 × 10^−14^	-	[33]
